# Guiding principles for evaluating the impacts of conservation interventions on human well-being

**DOI:** 10.1098/rstb.2015.0103

**Published:** 2015-11-05

**Authors:** Emily Woodhouse, Katherine M. Homewood, Emilie Beauchamp, Tom Clements, J. Terrence McCabe, David Wilkie, E. J. Milner-Gulland

**Affiliations:** 1 Wildlife Conservation Society, 2300 Southern Boulevard, Bronx, NY 10460, USA; 2 Department of Anthropology, University College London, 14 Taviton Street, London WC1H 0BW, UK; 3 Department of Life Sciences, Imperial College London, Silwood Park Campus, Buckhurst Road, Ascot SL5 7PY, UK; 4 Department of Anthropology, University of Colorado, 233 UCB, Boulder, CO 80309-0233, USA

**Keywords:** development, impact evaluation, livelihoods, poverty, social impact, well-being

## Abstract

Measures of socio-economic impacts of conservation interventions have largely been restricted to externally defined indicators focused on income, which do not reflect people's priorities. Using a holistic, locally grounded conceptualization of human well-being instead provides a way to understand the multi-faceted impacts of conservation on aspects of people's lives that they value. Conservationists are engaging with well-being for both pragmatic and ethical reasons, yet current guidance on how to operationalize the concept is limited. We present nine guiding principles based around a well-being framework incorporating material, relational and subjective components, and focused on gaining knowledge needed for decision-making. The principles relate to four key components of an impact evaluation: (i) defining well-being indicators, giving primacy to the perceptions of those most impacted by interventions through qualitative research, and considering subjective well-being, which can affect engagement with conservation; (ii) attributing impacts to interventions through quasi-experimental designs, or alternative methods such as theory-based, case study and participatory approaches, depending on the setting and evidence required; (iii) understanding the processes of change including evidence of causal linkages, and consideration of trajectories of change and institutional processes; and (iv) data collection with methods selected and applied with sensitivity to research context, consideration of heterogeneity of impacts along relevant societal divisions, and conducted by evaluators with local expertise and independence from the intervention.

## 1. Introduction

In response to both evidence of the dependence of vulnerable human populations on ecosystems [1] and the costs associated with some conservation interventions for local people [2,3], the question of how to reconcile conservation with human development has emerged as a key policy issue [4]. Recognition of the inadequacies of narrow economic indicators such as income and consumption in measuring social development has shifted attention to a broader and multi-faceted vision of human well-being [5]. For instance, a conservation intervention may improve the local economy by providing jobs and alternative livelihoods, but could negatively affect other priorities for local communities such as social relationships or autonomy. Where the specifics of social impacts are not intentionally and systematically examined, they could be misunderstood or missed entirely, with repercussions for social justice and conservation outcomes. While there is ready acknowledgment in the environmental literature that robust empirical evaluation is required in order to better understand which approaches can work for biodiversity [6], extension of this premise to conservation impacts on human lives is still rare. Across a range of conservation strategies, there is a lack of evidence of the impacts on human well-being that adequately capture the complexity on the ground [7].

Despite the variety of definitions, there is increasing agreement in international policy on a conception of human well-being that encompasses objective material circumstances of people's lives such as housing, income, livelihoods, health and the environment, social aspects such as community networks, and a subjective component capturing an individual's assessment of their own circumstances [5,8]. Well-being can therefore be defined as a positive physical, social and mental state [9]. Understanding the impacts of conservation on the multiple dimensions of people's lives and working to improve them are ethical imperatives for conservation practitioners, as well as being important to the success of strategies. Conservationists have responsibilities towards the communities they work in, to ensure at the very least they do not harm people [10], a premise that is encapsulated in policy commitments such as the Durban Accord on protected areas [11]. Well-being is also important for policy analysis, because its pursuit is a primary driver of people's decision-making [12]. Interventions that support local well-being can increase environmentally desirable behaviour, and lead to positive local perceptions and engagement [4]. Using a well-being framework provides a holistic way to incorporate goals for different values (e.g. livelihoods and the environment) into decision-making, which can also help to build political support and mobilize funding. For these reasons, it is vital that well-being is taken into account both in conservation programmes that explicitly incorporate livelihoods, and in those with narrower biodiversity targets.

Recent studies on the impacts of protected areas on poverty [13–15] have used robust quasi-experimental designs but have tended to focus on externally defined asset- and monetary-based measures, or on human–wildlife conflict and attitudes towards parks [16]. Further, by only measuring average net impacts, studies do not reveal how benefits and losses are distributed across different groups of people. With the recent exception of a more nuanced evaluation of marine protected areas (MPAs) [17], these types of studies do not consider multiple dimensions of well-being and its subjective aspects. Crucially, much research does not elucidate the mechanisms through which interventions impact well-being [18], an element of evaluation that is especially important for decision-making [19].

One difficulty in embarking on a well-being impact evaluation is in operationalizing such a wide and complex concept. The array of conceptual frameworks for well-being [20] can cause confusion, especially among researchers and practitioners trained in the natural rather than social sciences. Although the Conservation Measures Partnership [21] advocates inclusion of human well-being targets when designing and adaptively managing conservation projects, it provides no methodological guidance on how to measure and evaluate well-being impacts, and ignores the possibility of unexpected consequences, institutional changes resulting from interventions and the importance of local perspectives of change. Stephanson & Mascia [22] build upon this work with a broader conception of well-being, but do not consider subjective experiences, and remain focused on methods for collection of data relevant to conservation planning rather than impact evaluation.

We propose accepting the plurality of the concept of well-being, and present guiding principles based around existing theoretical frameworks, an approach that allows comparable but locally relevant results. Our principles are intended to support evaluators in operationalizing a multi-dimensional conceptualization of well-being to measure and understand impacts in ways that align with realities on the ground. We take a pragmatic approach to evaluation focused on gaining the knowledge needed for decision-making and policy development, a perspective that necessitates flexibility and an openness to mixed methods, incorporating quantitative and qualitative data and analysis [23]. Our aim is for the principles to be useful for conservation practitioners and adaptable to small-scale projects with limited budgets and technical expertise, as well as larger programmes that have research capacity. We do not advocate the use of particular tools but aim to guide critical thinking in applying methods in ways that support depth of understanding and robust results appropriate to the evidence required. The principles relate to four key stages of conducting an impact evaluation found in the literature [24–26], and take into account some of the challenges to evaluation in conservation such as nonlinear response outcomes, lack of comparators, multiple outcomes and complex confounding factors [24,27]. The four stages are: (1) defining outcomes of interest and well-being indicators, including formulating complex theories of change and considering confounding factors; (2) designing the evaluation to link outcomes to the intervention; (3) understanding processes of change; and (4) collecting data on selected indicators and contextual factors. Step 3 reflects the increasingly recognized need in conservation policy for richer understandings of mechanisms through which impacts are produced [18].

## 2. Conceptualizing human well-being

In conceptualizing well-being, there is a tension between a universal approach that allows comparisons, and ensuring local relevance [28]. Local perspectives must drive our understanding of well-being, as externally derived categories may not have meaning for local people, and thus will not account for locally significant impacts of interventions. Any universal frameworks or methods used in evaluations must be adaptable to locally meaningful formulations, made relevant to the target population by using comparable categories with locally specific indicators. There is also a balance to be struck between objective and subjective definitions. In development economics, well-being has been conceived of as an objective concept mainly focused on material assets, and in social psychology as an internal, subjective psychological state felt by the individual. Using either approach alone is insufficient. People will have different capabilities to gain benefits from assets, whereas a person's expressed satisfaction with life is a poor guide to objective valuation of material impoverishment, as it does not account for people adapting their preferences to harsh conditions [29]. Conservationists should be interested both in objective indicators, as they show tangible changes and are often most sought by funders and policy-makers, and also in subjective well-being, because people's feelings and experiences impact on participation and social sustainability.

One framework that combines objective and subjective valuation and gives primacy to local understandings comes from the Wellbeing in Developing Countries (WeD) project [30]. Well-being is conceptualized as an outcome and a process, in three interacting dimensions: the objective material circumstances of a person, subjective evaluation of people's goals and the processes they engage in, and a relational component [31]. This last dimension acknowledges that individual well-being is pursued in relation to other people, that social connectedness is a human need and that definitions of a good life are socially constructed [32]. Culture is often viewed as external in discussions on poverty and well-being, but here it forms the lens through which all aspects of well-being are constituted [33]. For example, the significance of cattle-raising goes beyond a livelihood for pastoralist communities to being a culturally meaningful way of life entailing social contracts of ownership or use rights over land. The WeD approach emphasizes the holistic, dynamic and social nature of well-being. It brings together a unique configuration of interdependent elements, counterbalancing a tendency in policy to privilege material well-being and underplay subjective feelings and the social dimension of people's lives [31].

Another framework—the ‘Voices of the Poor’ (VoP)—is based on empirical data and is familiar to conservationists, because it was used in the Millennium Ecosystem Assessment as a means of conceptualizing relationships between ecosystem services and aspects of well-being. The project found five components commonly considered to constitute well-being among individuals across 23 countries [34]. They are material assets, health, social relations, security and freedom of choice and action. The last component, which underpins the others, means having a sense of control over one's life and the capacity to achieve what one values doing and being. This is easily overlooked in conventional assessments but may be especially relevant for conservation interventions, which can be rejected if perceived as imposed and undermining freedom with regard to environmental behaviour [35]. On the other hand, interventions that secure local land tenure and improve natural resource governance could increase feelings of empowerment [17].

The VoP framework provides a useful checklist of themes to consider when starting an evaluation, based on empirical research. We combine it with ideas from the WeD research, thus providing a thematically based framework that allows depth of understanding, and can be used to guide the structure of evaluations (table 1). The VoP domains will be informative to evaluators about which aspects of well-being to consider, bearing in mind that WeD's three-dimensional perspective will help to define the questions asked and the type of data collected. For example, in studying the benefits of a payment for ecosystem services programme, in the material domain, a relevant (and commonly used) objective indicator could be household income. The subjective dimension suggests consideration of levels of satisfaction with income changes, and feelings of fairness about benefit distribution. The relational dimension suggests the relevance of how people use income, the way it can change relationships and differential capabilities within a household to benefit.

**Table 1.** Theoretical framework for well-being evaluation, which links ‘Voices of the Poor’ (VoP) well-being domains with perspectives from Wellbeing in Developing Countries (WeD).

‘Voices of the Poor’ well-being domains	description and examples	insights provided by WeD perspective and research
material	secure and adequate livelihoods enough food and food security assets, e.g. land, natural resources, livestock, savings and capital, goods, housing, furniture and tools	not only about what people have, but what they can do and be, and how they feel about these things the ways in which objective material well-being outcomes are defined and satisfied are socially and culturally constructed, requiring attention to local context human as well as material resources are important, including knowledge and education
health	feeling strong and well access to health services appearing well having a healthy physical environment e.g. fresh air	health is subjectively experienced mental health is as significant as physical health in well-being
social relations	good relations with family, community and country dignity, e.g. not being a burden, feeling listened to ability to help others and fulfil social obligations ability to care for children (including education and marriage)	collective well-being is significant for individual well-being in culturally defined ways social structures and institutions that enable people to pursue well-being in relation to one another may be impacted by interventions people's ideas and strategies for pursuing well-being may not be compatible, resulting in trade-offs that must be confronted
security	confidence in the future—predictability, peace safe and secure environment, e.g. safety from disasters personal physical security and safety security in old age and for future generations	people's well-being and decisions are influenced by perceptions of future and perceived threats capabilities to achieve other aspects of well-being may increase security sustained security can only be the outcome of autonomy rather than dependency
freedom of choice and action	sense of control and power ability to pursue what you value doing and being, and meet aspirations ability to be a good person, e.g. to help others	not about independence but self-endorsement of one's own behaviour, i.e. feeling personal value and interest regarding actions autonomy can be evaluated with regard to different aspects of people's lives that they value related to the ability to adapt in times of change

## 3. Principles to guide well-being evaluation

We next discuss the nine key principles to bear in mind when carrying out a robust evaluation based on the framework presented in table 1. These principles are summarized in table 2.

**Table 2.** Summary of guiding principles for evaluating impacts of conservation interventions on well-being.

guiding principle	examples of approaches to addressing the principle	references for further details
defining outcomes and indicators		
(1) put local people at the centre of the analysis	start with flexible qualitative research, e.g. semi-structured interviews to explore local understanding and components of well-being	[35,36]
	qualitative research on the local context, e.g. through literature reviews and informed sources	
	map out theory of change developed with participating communities and stakeholders	[21,25,37]
(2) select multiple outcomes to measure and consider subjective components	select multiple well-being indicators based on local priorities and outcomes in theory of change	
	collect qualitative data on outcomes not amenable to quantification, e.g. institutional change	[38]
	collect data on subjective feelings about pertinent aspects of well-being	[39]
	allow opportunities for people to voice unintended consequences, and negative outcomes	
	consider relationships (trade-offs and synergies) between outcomes	[40]
	evaluate impacts on security through identifying locally relevant indicators	[41]
evaluation design		
(3) match evaluation design to the setting and questions asked	consider quasi-experimental and before-after-control-intervention designs	[14,15,26,42]
	if no baseline data, consider recall interviews for simple variables	[43]
	control-intervention designs without baselines should be supported by other data	
	alternatives to quasi-experimental designs: theory based, case studies, participatory methods	[19,37,44,45]
understanding processes of change		
(4) provide evidence of causal linkages	theory-based analysis using quantitative and qualitative methods to understand the how and why of impacts	[36,37,46]
	quantitative data can produce estimates of the contribution of different causal mechanisms	[18]
(5) consider trajectories of change	anticipate and acknowledge possible trajectories of change and measure ex-post impacts if possible	[17,47,48]
(6) investigate institutions and governance structures	institutional analysis using secondary and primary data	[49]
	participatory institutional profiling (before-and-after intervention)	[50]
data collection		
(7) select and apply methods with sensitivity to context	choose tools appropriate to the cultural context and apply with consideration to equity combine methods to take advantage of their strengths, e.g. quantitative measures with qualitative insights	[51,52]
(8) take into account heterogeneity	disaggregate data according to qualitative understandings of social structures and livelihoods	[53,54]
	individual interviews to capture differences across age and gender within households	[39]
(9) ensure independence	recruit locally trusted people independent of implementing institutions and conservation	[36]
	draw upon local language skills and in-country researchers	

### (a) Defining outcomes and indicators

#### (i) Principle 1: put local people at the centre of the evaluation

One of the central benefits of using a broadly defined, locally grounded conception of well-being is that it puts at the centre those people most affected by policy changes and interventions. Local people should be involved throughout the process of evaluation, but most crucially when initially defining the scope of the evaluation. Interventions are based on a theory of change (ToC), which explains the process through which the intervention is thought to give rise to specific outcomes. An evaluation is effectively testing this theory. To start it is helpful explicitly to map out the ToC causal chain from inputs to outcomes, the underlying logic and the social, behavioural and institutional assumptions being made [21,25], a process that allows space for reflection on assumptions and context, and the development of hypotheses and evaluation questions. External drivers and pressures such as government policies, market changes, climate change and environmental shocks, and how they may be changing well-being and interacting with the intervention, must be considered in order to take confounding factors into account. A ToC is best developed with the participation of local stakeholders who hold highly contextual knowledge, and may well consider potential consequences and unintended changes that would not otherwise be addressed. For single-stranded projects, tracing potential changes may be relatively straightforward, but for complicated projects with multiple components linked together (e.g. land rights, education and livelihoods), more thought must be given to interactions and feedback loops [46].

Qualitative research, using semi-structured, informal interviews and participant observation, and which is flexible and open to unexpected findings, can provide details on local nuances in the language used to express well-being, and the priorities and aspirations that people have. For example, Abunge *et al.* [35] used focus group discussions with different stakeholder groups (e.g. women fish vendors and beach seine captains) connected to a Kenyan coastal fishery to understand how well-being was expressed by different types of people, how and why it had changed, and the hopes people had for the future of the fishery. Open-ended questions such as ‘How would you describe in general a person who is doing well in this community?’ encouraged people to open up and discuss what constituted a good life in this particular context. Qualitative research is also valuable at the start of evaluation to understand the historical, political and cultural issues that can shape people's perceptions, helping in the development of locally relevant questions for any structured and standardized questionnaires [36]. Other useful sources of insight are past studies, ethnographies and informed sources who understand local politics and history.

#### (ii) Principle 2: select multiple outcomes to measure and consider subjective components

Given the multi-dimensional nature of the well-being conceptualization presented here, it cannot be captured by measuring only one outcome. Rather than using a standard list of indicators that may be irrelevant, evaluators can target pertinent components suggested during qualitative discussions with communities and considered sensitive to the intervention actions in the theory of change. The framework in table 1 can be used to guide this inquiry. For example, health and nutrition may be prioritized in the community and could be improved by access to water and food sources, or social capital could be increased through the establishment of community-based governance of natural resources. Quantitative indicators could then include number of meals eaten per day or levels of participation in community activities. Some outcomes relevant to well-being, such as social relations or political change, may not be amenable to quantification at all [38]. Although the concept of well-being takes a more positive perspective than the concept of poverty, it is also important to include negative information when assessing interventions. External interventions may contribute as much towards ‘ill-being’ e.g. social exclusion, conflict or malnourishment, as to well-being [34].

The need for measurement of multiple outcomes of interventions is highlighted by the fact that there may be trade-offs and synergies between outcomes [40]. For instance, there could be trade-offs between different dimensions of well-being such as wealth and equity, invalidating conclusions about the overall impact of interventions on well-being if one dimension was missed. Considering these relationships in developing a theory of change, and later in data analysis, moves the evaluation away from simplistic narratives towards a realistic acknowledgement of potential gains and losses that can inform decision-making. It can also highlight aspects of well-being that people feel cannot be ‘traded-off’ at all, such as cultural heritage [55].

Observable, quantitatively measured changes can provide credible evidence of impact to external audiences, but perceived change by local communities—reflecting the subjective aspect of well-being—may also be significant, especially for conservation managers on the ground wanting to take mitigating measures to improve elements of the project people are not satisfied with, in order to gain local support. Indeed, perceived well-being may be at odds with objective measures, highlighting where there is dissatisfaction with interventions. In villages involved in MPAs in Indonesia, for example, there were negative changes in perceived well-being despite increases in wealth (based on material assets) during the course of the intervention, owing to inequitable sharing of benefits, conflict and unmet expectations [17].

Security—living under conditions where there is predictability—is a key constituent of well-being (table 1), drawing attention to temporal aspects of well-being. People's current well-being takes place in the context of past experiences, as well as expectations, fears and aspirations about the future. People engage in trade-offs through time to establish security and reduce threats [41]. Especially when faced with rapid changes, perhaps as a result of an external intervention, uncertainty prevails, reducing security and therefore well-being. Conservation may increase feelings of insecurity, even if implemented in the hope of improving environmental security in the longer term. For example, in Tanzania concerns about future land-use restrictions were highest among households near to Tarangire National Park, and this influenced decisions to convert land to agriculture to secure land tenure, ultimately affecting conservation outcomes [56]. Alternatively, threats of large-scale land acquisitions in countries where community tenure rights are weak may lead to higher participation in conservation activities, such as seeking community forestry titles [57]. Evaluating impacts of interventions on security could involve developing indicators relevant to the context, such as livelihood and income diversity, access to justice, and the functioning and membership of collective institutions [41].

### (b) Evaluation design: linking outcomes to the intervention

#### (i) Principle 3: match evaluation design to the setting and questions asked

At the heart of evaluation is the process of attributing specific effects, in this case changes in well-being, to the intervention rather than to other factors. This can be achieved by inferring the counterfactual—what would have happened in the absence of the intervention—through the identification of controls, thus reducing bias from confounding factors [24]. Quantitative experimental and quasi-experimental designs that allow assessment of the magnitude of impacts are often viewed as the ‘gold standard’ for answering the attribution question in a robust way in development and conservation. Truly experimental designs, where the researcher randomly assigns interventions, are not often possible in conservation [6], and instead quasi-experimental methods are used in which control groups (most likely households or villages) are identified through matching techniques [26]. Controls are selected based on their similarity with the intervention targets on a suite of measurable covariates that are thought to affect participation in the intervention and the outcomes of that intervention (according to the theory of change). For example, in the case of protected areas, these covariates could be distance to a city, elevation and asset-based indicators [15]. Controls should be selected outside the zone of influence of the intervention, to avoid spillover effects of the project or contamination by other interventions. Clements & Milner-Gulland [42] evaluated livelihood outcomes of protected areas in Cambodia, and selected control villages based on matching variables thought to affect village-level poverty and access to natural resources prior to the intervention. They ensured that control villages were more than 20 km from the protected area border. It is important, however, to recognize that controls in that study did not reflect the groups to which people actually compared themselves, which could affect subjective well-being and therefore interpretations of impact. A more qualitative approach to matching could instead be used, by selecting controls with participation from local people, to identify controls that are both methodologically appropriate (i.e. deal with confounding factors) and meaningful for local people (e.g. geographically closer). It is also worth considering that there may be heterogeneity within the treatment, such as spatial differences in the impact of an intervention that will be disguised if only average effects are considered. For example, people experienced the same alternative livelihood project very differently across different villages in Tanzania [58].

Using baselines in addition to controls to create a BACI (before-after-control-intervention) evaluation allows impacts to be isolated from two biases: selection bias in which the targets of the intervention are a non-random selection of the whole population (for example, when wealthier families are more equipped to participate in a PES (payment for ecosystem services) scheme), and concurrent change (for example, improvements in wealth affected by both a sustainable livelihoods intervention and wider economic changes) [6]. Selection bias is tackled by matching techniques in quasi-experimental designs, while tracking change through time in both the control and intervention (the difference in difference method) takes into account differences between treatments and controls that are constant over time, including unobserved intrinsic characteristics such as levels of motivation or optimism. A prospective evaluation, developed at the same time as the intervention is designed, is preferable to a retrospective evaluation as it allows the collection of baseline data and is more likely to generate valid counterfactuals [26]. It is possible, however, to construct an approximate baseline ex-post from secondary data or carefully designed surveys asking participants to recall specific, easily remembered variables such as assets and link them to locally important events [59], although researchers should be aware that recall data are prone to inaccuracies [43]. Although before–after and control–intervention comparisons on their own make for weaker causal inferences, where a full BACI design is not possible, these simpler designs can provide credible insights if supported by other data such as community perceptions that substantiate or refute the quantitative trends.

There are real-world constraints to using controls and quasi-experimental designs to attribute changes to an intervention; they require a large sample size, may not be suitable for complex or broadly defined interventions such as regional policy with small number of units, and require a suitable budget and technical skills [19]. There may be ethical concerns such as raising expectations or subjecting people to surveys that bring no benefits [51]. Controls may be difficult to find if, for instance, the intervention focusses on small areas of particular importance for conservation that are very different from the rest of the region (e.g. islands of natural forest). Instead of, or in addition to, quasi-experimental designs there are a range of alternative approaches through which causal inferences can be made that can be divided broadly into three types: theory-based, case studies and participatory. These methods tend to be stronger than quantitative counterfactual approaches in explanation and contextualization, but weaker on estimating the magnitude of impacts [19]. Decisions about which design to use must be made on the basis of resources and skills available, and the types of evidence required.

Theory-based methods consider the likely chains of impact, presenting alternative hypotheses for change and examining them through both qualitative and quantitative data. These can highlight where there is a break in the causal chain to explain impacts of an intervention [46]. A case study approach focuses on particularly successful (or unsuccessful) cases (e.g. villages or intervention types) to examine the contribution of the intervention to outcomes. Comparisons across the cases can elucidate the combinations of causal factors (the types of intervention, methods of implementation such as levels of capacity building, and contextual factors such as tenure regimes, wealth levels and local leadership) explaining changes in well-being indicators [44]. Participatory methods allow communities to systematically assess changes themselves; for example, group discussions, in which causes for reported changes in well-being are ranked and scored, can show relative perceived impacts of intervention and non-intervention factors, with greater levels of agreement between groups indicating reliability [45]. The use of ‘reflexive counterfactuals' where participants compare themselves before and after the intervention, by prioritizing perceptions, may be subject to bias, but provide important information, for example, in protected area management focused on improving equity and effectiveness at the site level [60].

Given the pros and cons of the quasi-experimental and alternative approaches to evaluation design, Roe *et al.* [61] advocate a sensible two-track system, in which in-depth longitudinal evaluation using controls for a selection of representative interventions of strategic relevance is combined with rapid, participatory assessment more feasible for the majority of projects. Policy-makers and donors may emphasize the former approach, to gain evidence of the magnitude of impact needed for cost-effectiveness analysis. This may guide decisions on whether to replicate or fund similar interventions in the future. Field managers wanting to understand people's experiences and perspectives may focus on the latter, combined with quantitative indicators analysed through theory-based analysis.

### (c) Understanding processes of change

#### (i) Principle 4: provide evidence of causal linkages

Using a counterfactual approach that is limited to attributing outcomes to an intervention cannot answer the fundamental questions of *how* and *why* a project is or is not effective, and how contextual factors may be hindering or reinforcing change in particular outcomes [62]. This is especially important where evaluation is directed towards lesson learning. Theory-based approaches as described above take a deductive approach by empirically discounting alternative plausible explanations for outcomes. For example, a theory-based analysis showed that an infant nutrition project in India mis-targeted mothers who, although gaining knowledge, were not able to put it into practice as it was their mothers-in-law and husbands who made decisions about food and child-raising, an insight that was initially found through reading anthropological studies [37]. In that example, it was a design flaw in the project that led to poor results, but a theory-based approach can also detect problems with implementation. Ferraro & Hanauer [18] demonstrate how a quasi-experimental design can incorporate quantitative analysis of causal pathways to show how protected areas in Costa Rica reduced poverty mainly through tourism. The fact that the three measured causal mechanisms accounted for only two-thirds of impacts in this study serves to highlight how theories of change must incorporate in-depth and complex understanding of socio-ecological systems to fully capture processes of change.

#### (ii) Principle 5: consider trajectories of change

Well-being is not a discrete outcome, but an ongoing dynamic process, changing through the course of an intervention and beyond [63]. Trajectories of change are not linear, resulting in attribution errors if well-being effects of an intervention are measured at only one point in time [47]; for example, there could be high initial impact owing to improved forest governance arrangements that is eroded through time by pre-existing power structures. Monitoring throughout the course of a project is ideal as it allows real-time feedback for learning and adjustment [62]. Ex-post assessments are rare, but may be crucial in understanding longer-term impacts and sustainability, and for taking into account time lags between intervention and effect. For example, any initial gains in aspects of well-being such as fish catch, wealth and empowerment were lost after external support for MPAs in Indonesia was withdrawn, suggesting that interventions of this kind need to build capacity, gain broad-based support and sustain funding [17]. Realistically, it may only be possible to evaluate shorter-term outputs or outcomes and indicate where longer-term impacts could occur [25]. The reference standards of those affected by interventions can also change, potentially as a result of the interventions themselves [48]; for instance, increasing material wealth may lead to wealth becoming a more important component of well-being for some, but still reduce well-being owing to rising aspirations not matched by opportunities [64].

#### (iii) Principle 6: investigate institutions and governance structures

Well-being depends on institutions—human-devised informal constraints and formal rules—which govern relationships between individuals and groups, and between humans and ecosystems [65]. The choices made about the types of organizations conservationists work with (state agencies, private corporations, customary authorities) will profoundly shape the institutional landscape, affecting representation, citizenship, and ultimately social and environmental sustainability [66]. Ill-considered interventions can subvert existing institutions and cultural practices that act to regulate environmental behaviour, resulting in alienation and counter-productive conservation results [67]. Natural resource management interventions can, on the other hand, support improved governance, contributing to poverty alleviation [68], and act as vehicles for social change and improved participation. This emphasizes the relational dimension of well-being, and the attention required in analyses to the relationship between individual and collective well-being, which is shaped by dynamic institutions such as norms. During evaluations, it is important to understand the functioning of institutions and governance structures acting within and upon communities, and how conservation interventions affect it, in turn impacting individual well-being. This can be achieved through, for example, institutional profiling with local people in which visual methods such as Venn diagrams can aid discussions about key institutions, relationships and forms of power at different scales that influence people [49,50].

### (d) Data collection

#### (i) Principle 7: select and apply methods and toolkits with sensitivity to the research context

There are many tools and methods available—both with a quantitative and qualitative slant—which conservation evaluators can draw upon in collecting data on well-being indicators as well as on contextual and confounding factors. However, reflection on their appropriateness to the context is vital for data validity. Schreckenberg *et al.* [51] provide a useful compilation of rapid social research methods that can be used to collect data on the well-being impacts of conservation interventions. These methods are compatible with our proposed framework for well-being evaluation, but thought needs to be given not only to selecting particular methods appropriate to the context, but also to the ways these tools are applied to deal with culturally sensitive issues, vested interests and equity. For example, how will the use of particular local informants skew the evaluator's understanding of the issues, and how will marginalized groups be accessed? Tallying scores for different outcomes, as advocated in some guidelines [69], is attractive for quick and standardized assessments. But, in isolation, scoring systems run the risk of aggregating over a broad range of indicators and social groups. This may lead to their falling between two stools; meaningless both locally and comparatively. Even where carefully selected and appropriately applied, a single method may be inadequate on its own. For example, the Basic Necessities Survey (BNS) is an index-based tool that assesses household poverty based on locally defined ‘necessities' [70]. A key benefit of the method is that it produces quantifiable results through a participatory and democratic process, but the score is aggregated at the household level and gives little detail about the processes of change. A hybrid research design augmenting the BNS with data that chime with WeD's broader conception of well-being could form a more robust way to capture the complexity of well-being change. For example, the incorporation of semi-structured interviews could focus on subjective experiences, and capture causal mechanisms.

The methods of Participatory Rural Appraisal (PRA) used by development practitioners have been enthusiastically adopted by many conservationists, to provide locally co-produced data relatively quickly and cheaply, and to demonstrate local involvement in evaluation. Relevant methods include qualitative resource mapping, timelines, focus groups, village histories, ranking and scoring [45]. The mainstreaming of PRA, however, has resulted in the process becoming somewhat ritualized, and the participatory label is often used to mask standard extractive data collection. Participatory discussions can be susceptible to co-option by local elites, silencing those most affected [71]. If PRA techniques are used, experienced and trained facilitators familiar with the local context, and independent of the intervention, should lead the work to ensure sensitive and equitable discussion [52]. Groups should be appropriately constituted; for example, different gender and age groups may not be comfortable in mixed groups. In some situations, for example, where communities are suffering from research fatigue, or issues are highly contested, individual or household interviews may be a better option.

#### (ii) Principle 8: take into account heterogeneity within the target group

Just as different people are able to access different ecosystem services, there will be trade-offs between the well-being impacts of interventions on different people, between or within communities [53]. Standard experimental and quasi-experimental impact evaluation methods may produce an average effect of an intervention across households, communities or the whole population being investigated, but this does not address which types of people win and lose, and why [72], unless heterogeneity is purposefully incorporated into research design and data collection. It is especially important to ensure that vulnerable groups such as the poorest, landless, migrants or mobile resource users (such as fishers, pastoralists and forest dwelling groups) are included in evaluations as they are often invisible unless local knowledge is used [73]. The impacts of interventions on households with different livelihoods may differ significantly; for example, non-timber forest product collectors in Cambodia were significantly better off in terms of basic necessities inside a protected area than outside owing to secure access to resources [54]. Impacts of interventions on social dynamics between different groups can undermine well-being; for example, targeting only some groups may create conflict [38]. Qualitative research can elucidate social structures, wealth and livelihood differences that can form the basis for appropriate disaggregation of data, as well as improving understanding of the ways different groups conceive of well-being.

Social surveys are often carried out at the household level, but there is likely to be intra-household variability, with differences in well-being according to gender and age. Britton & Coulthard [39] found that the domains of life important for well-being, and satisfaction with these domains, differed significantly between men and women in fishing communities in Northern Ireland. Women may lose resource access under payment-based conservation interventions but receive few of the benefits, which are given to male household heads [74], or they may be excluded altogether from participation [75]. Well-being is experienced by individuals, and so they should be the primary unit of impact assessment rather than the household, which is a common evaluation unit in economic assessments. However, as the WeD framework suggests, individual well-being is relational. Therefore, collective well-being at different scales of social relationship is significant for individual well-being, although the extent and nature of these relationships will differ across cultures and contexts [63].

#### (iii) Principle 9: ensure independence

Although the research design and selection of methods and techniques are important elements to consider in an evaluation, the quality of research will ultimately be defined by how it is conducted and the relationships established between the researchers and participants [76]. Evaluations conducted by people who are independent of actors implementing interventions or otherwise working in the system will result in improved validity. Although research is often labelled as ‘independent’, it is all too often facilitated by vehicles clearly marked with government or NGO logos, or by people who are linked to the intervention or to conservation more generally. Marginalized rural people are likely to find it difficult to talk candidly with powerful individuals, which suggests the importance of considering local language, trust and ethnicity in building a research team. Calling upon local expertise and language skills will decrease the risk of obscuring local meaning and realities [36]. In-country students or young researchers supported by experienced evaluators may be good options for tight budgets, and this approach will also contribute to capacity building within the country.

## 4. Conclusion

Understanding the intricacies and dynamics of what people consider to be a good life is far from straightforward. Well-being is multi-faceted, and varies between contexts and cultures, within communities and households, and through time. Attributing well-being change to interventions must also take place in the context of complex and dynamic influences at multiple scales. This may seem like an impossible task for conservation practitioners, especially with limited resources and expertise. In the face of this complexity, however, formulaic methods will not work. Conservationists should not use prescriptive designs and methods without thinking about their applicability to the case, how best to apply to them and what sort of data they will produce. By engaging with the principles and concepts set out here, and summarized in table 2, conservationists can hope to untangle the complexity of social impact evaluation, and improve their understanding of objective and subjective well-being impacts. This understanding is perhaps most urgently needed in materially poor areas of the Global South, but well-being provides a useful way to measure social impacts of conservation regardless of the wealth status of the population [77]. Mixed methods can better support causal and explanatory analysis, and conservation researchers should not be reticent about using qualitative data in their own right. Far from signaling a lack of rigour, qualitative approaches are necessary to appropriately disaggregate data, identify covariates, explain the processes involved in producing well-being impacts, and allow local voices to be heard. The important basis for rigour is the appropriate application of techniques, either quantitative or qualitative [78].

The evidence base on the impacts of conservation interventions on human well-being is weak, and there is much to be learnt to support decision-making about the range of interventions used in conservation under different contexts. For example, in a recent systematic review of 136 community-based conservation evaluations, 80% of the studies included were rated as poor quality on the basis of conflict of interest (i.e. lack of independence), data validity and other problems [79], arguably throwing the results of an otherwise meticulous statistical analysis into serious doubt. There will inevitably be trade-offs between conservation outcomes and human well-being outcomes [80], and between different elements of well-being itself. Only by assessing well-being in a way that tackles complexity, context, politics and the wide range of impacts that conservation can bring, can stakeholders hope to openly discuss and negotiate trade-offs in a systematic and transparent way. Conservationists have a responsibility to the communities in which they carry out their activities, and using well-implemented well-being evaluations can improve accountability and lesson learning, ultimately improving the likelihood of successful, locally supported conservation in the long-term**.**
